# ‘Boom‐and‐busted’ dynamics of phytoplankton–virus interactions explain the paradox of the plankton

**DOI:** 10.1111/nph.18042

**Published:** 2022-03-09

**Authors:** Kevin J. Flynn, Aditee Mitra, William H. Wilson, Susan A. Kimmance, Darren R. Clark, Angela Pelusi, Luca Polimene

**Affiliations:** ^1^ 61564 Plymouth Marine Laboratory Prospect Place, West Hoe Plymouth PL1 3DH UK; ^2^ 2112 School of Earth and Environmental Sciences Cardiff University Cardiff CF10 3AT UK; ^3^ Marine Biological Association of the UK, The Laboratory Citadel Hill Plymouth PL1 2PB UK; ^4^ 6633 School of Biological and Marine Sciences University of Plymouth PL4 8AA UK

**Keywords:** competition, cyst, paradox of the plankton, phytoplankton, succession, virus

## Abstract

- Rapid virus proliferation can exert a powerful control on phytoplankton host populations, playing a significant role in marine biogeochemistry and ecology. We explore how marine lytic viruses impact phytoplankton succession, affecting host and nonhost populations.
- Using an *in silico* food web we conducted simulation experiments under a range of different abiotic and biotic conditions, exploring virus–host–grazer interactions and manipulating competition, allometry, motility and cyst cycles.
- Virus‐host and predator–prey interactions, and interactions with competitors, generate bloom dynamics with a pronounced ‘boom‐and‐busted’ dynamic (BBeD) which leads to the suppression of otherwise potentially successful phytoplankton species. The BBeD is less pronounced at low nutrient loading through distancing of phytoplankton hosts, while high sediment loading and high nonhost biomass decrease the abundance of viruses through adsorption. Larger hosts are inherently more distanced, but motility increases virus attack, while cyst cycles promote spatial and temporal distancing.
- Virus control of phytoplankton bloom development appears more important than virus‐induced termination of those blooms. This affects plankton succession – not only the growth of species infected by the virus, but also those that compete for the same resources and are collectively subjected to common grazer control. The role of viruses in structuring plankton communities via BBeDs can thus provide an explanation for the paradox of the plankton.

## Introduction

An enduring question in aquatic ecology is centred on the so‐called ‘paradox of the plankton’ (Hutchinson, 1961) – why are there so many phytoplankton species, which are apparently constrained by the same limiting factors, inhabiting open water ecosystems? Competition for limiting resources typically leads to exclusion of species that outwardly appear uncompetitive, in both empirical and modelling studies (Hambright & Zohary, 2000; Passarge *et al*., 2006). Despite this, many dozens of species are found coinhabiting natural habitats. The existence of such populations (i.e. the paradox of the plankton), has been traditionally explained by invoking the careful selection of traits amongst the producers and consumers (Abrams, 2000; Huang *et al*., 2016) and for phytoplankton with roles for allelopathic agents (Fistarol *et al*., 2005; Felpeto *et al*., 2018). There are important impacts of numerical abundance in such interactions (Holt, 1984) because of the sensitivity of trophic dynamics to predator–prey encounter rates and subsequent nutrient recycling (Mitra & Flynn, 2006) An alternative explanation for coexistence employs a ‘kill‐the‐winner’ concept (otherwise known as the KtW hypothesis – Thingstad, 2000; Winter *et al*., 2010), which assumes that a density‐dependent grazing factor prevents over‐dominance by an individual group of organisms. However, while the KtW hypothesis may work on a theoretical basis, there is little evidence that such processes operate in reality, as grazers and allelopathic agents invariably have a wide prey range (Flynn & Mitra, 2016; Felpeto *et al*., 2018). Recently, Behrenfeld *et al*. (2021) suggested that the paradox of the plankton might actually be a question of why there are so few plankton species, not so many, given the multitude of micro‐niches present in surface ocean waters. Here we examine the role of viruses in controlling the dominance of individual species.

The existence of marine viruses has been known for over seven decades (reviewed by Breitbart, 2012). Viruses are suspected to play a significant role in marine biogeochemistry and plankton ecology via the viral shunt, particularly through their impact on the growth and production of numerically abundant bacteria and phytoplankton (Wilhelm & Suttle, 1999; Motegi *et al*., 2009; Sullivan *et al*., 2017). In marine research, most emphasis has been placed on lytic viruses that kill the host (e.g. Brussaard & Martinez, 2008); thus, here we consider the impact of these lytic viruses on phytoplankton population dynamics using an *in silico* simulator based on a recently developed model (Flynn *et al*., 2021).

We explored the potential for lytic viruses to provide an ecologically plausible species‐specific alternative to the hypothetical KtW concept, through what we term the ‘boom‐and‐busted‐dynamic’ (BBeD) hypothesis. This BBeD is quite different to the traditional ‘boom‐and‐bust’ dynamic, which gives simple cycles of growth such as predator–prey oscillations; BBeDs show continued suppression of organism success following a particularly large viral lysis event (hence busted). Through the BBeD mechanism, a species that manages to attain a particular level of success on one occasion, and is subjected to virus infection, effectively sows the seeds of its own control by loading the water column with viruses that prevent blooms of subsequent generations. This mechanism affects plankton succession, not only affecting the growth of the species infected by the virus, but also those other species in the system that compete for the same resources and are collectively subjected to grazer control.

We also consider the importance of allometry and cyst cycles as traits affecting these dynamics. Allometry affects virus adsorption onto particles (Murray & Jackson, 1992), burst size (Edwards *et al*., 2021) and predator–prey encounter rates (Flynn & Mitra, 2016). The production and hatching of resting stages, or cysts, provide a mechanism to increase the temporal distancing. Our understanding of the biology and ecological significance of cysts varies greatly between protist plankton types (Reid, 1978; Ellegaard & Ribeiro, 2018). For diatoms, cyst formation has been shown to give a clear advantage against virus‐induced mortality (Pelusi *et al*., 2021) separating the phytoplankton host from its virus for a sufficient period and allowing significant decay of the virus load. In nature, cysts for some phytoplankton can potentially survive for centuries buried in sediments, with good excystment success on resuspension (Ellegaard & Ribeiro, 2018). Such long lockdowns would inevitably suppress virus attacks, and perhaps *de facto* prevent them from being noticeable at all except in certain conditions. Here we consider how cyst cycles of different durations could benefit the host.

## Materials and Methods

**Fig. 1:**
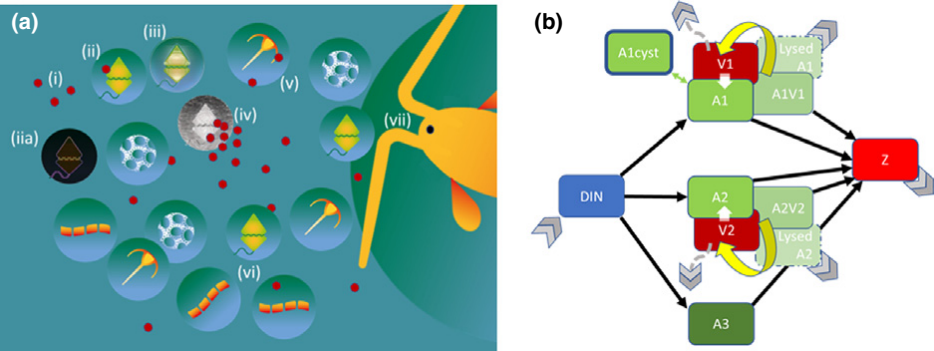
(a) Schematic illustration representing the ecological system. Virus plankton (i) encounters host cells (ii), with a proportion of encounters leading to infection (iii). Encysted host cells (iia) are considered immune to infection. After a latent period, the infected host bursts, liberating a very large number of viruses (iv). Some of these viruses will adsorb onto nonhost particles such as sediment, faeces, marine snow and other plankton (v), including competitors of the potential host (vi). The community is collectively subjected to grazing (vii), affecting the balance of host and competitors (vi), virus–host interactions (ii)/(iii)/(iv), and virus–nonhost (v) interactions. Not to scale with respect to abundance or size. (b) Schematic overview of the model. Three phytoplankton populations (A1, A2, A3) grow using dissolved inorganic nitrogen (DIN), and are grazed upon by zooplankton (Z). A1 and A2 are hosts for viruses V1 and V2, respectively; their infected subpopulations (A1V1 and A2V2) are also grazed upon by Z. A1 can encyst and excyst. Not shown are return flows of N to DIN from Z, and from the decay of faecal material, debris from bursts LysedA1 and LysedA2, and decreases in the abundance of viruses with decay and adsorption. Black arrows, main nutrient flows; yellow arrows, production of new virus cells from burst of infected host; green arrows, plankton–cyst exchange (for A1 only); grey chevron, removal/exchange of material from the upper mixed layer of the water column to underlying water which, for viruses, is also mediated via adherence to sinking particles (dashed grey lines).

An overview of the ecological interactions considered is shown in Fig. 1(a). The food‐web simulator (Fig. 1b) comprised three functional groups – virus, phytoplankton and zooplankton. The phytoplankton community consisted of three groups (A1, A2, A3), each of which could be characterised with respect to their cell size (equivalent spherical diameter, ESD), maximum growth rate (*µ*_max_) and motility. Additional information is provided in Supporting Information Methods S1.

In our study, A1 and A2 were set to represent species which were subjected to viral attack and had identical traits except that only A1 could be configured with the ability to form cysts; the *µ*_max_ of A1 and A2 were set at 1 d^−1^. For the cyst cycle, 0.1% of A1 production was encysted; excystment occurred at a given periodicity of 1 or 3 (lunar‐tide) months (Brosnahan *et al*., 2020 for other triggers). The viruses for A1 and A2 (V1 and V2, respectively) were also identical in their configurations (same size (50 nm); same adsorption coefficient, same minimum latent period of 0.5·host doubling time, burst size allometrically related to host as per Flynn *et al*., 2021). Thus, in the absence of cyst cycles, the dynamics of A1 and A2, their infected counterparts, and their viruses, were identical. Viruses were lost by adsorption onto any and all particles in the system (i.e. phytoplankton, zooplankton, debris of all forms and sediment; Murray & Jackson, 1992) but not onto each other, as well as by decay and mixing out of the mixed layer (Fig. 1); our previous study (Flynn *et al*., 2021) shows the potential importance of these losses on virus–host dynamics.

A3 was configured to grow with a maximum growth rate of 90% of that of A1 and A2; it was not subjected to viral infection. In the absence of viral attack on A1 and A2, A3 was thus rapidly excluded from the system as it grew more slowly. The role of A3 in the food‐web simulator was to describe the presence of all ‘other phytoplankton’ species as a functional group rather than any single species. A3 thus provided a pool of competitors against which to judge the competitive success of A1 and A2.

The growth of all phytoplankton was limited by the availability of dissolved inorganic nitrogen (DIN) and by light (which was affected by surface irradiance, and the light adsorption within the mixed layer due to light attenuation by the total phytoplankton biomass). Light was supplied with a surface photon flux density (PFD) of 2000 µmol m^−2^ s^−1^ with a 16.8 h : 7.2 h, light : dark cycle. The mixed layer depth was set at 10 m.

All phytoplankton types (A1, A2, A3), including their infected forms (A1V1, A2V2), were consumed by a common grazer, zooplankton Z. Like A3, Z was configured to represent a functional group rather than any single species. Grazing was configured according to biomass‐specific encounter rates which related to prey biomass and motilities (motility increased encounter rates) with no prey discrimination. Regenerated nutrients from zooplankton activity directly entered the DIN pool.

Nutrients were cycled from the decay of debris back to DIN via implicit bacterial activity. Mixing (with an effective dilution rate of 0.025 d^−1^) introduced DIN into the surface mixed layer and removed residual DIN and all forms of particulate materials. Also mixed into the surface waters was a low host‐specific virus load of 1 virus particle m^−3^; this prevented effective extinction of the virus at extreme low host abundance.

The mathematical construct of the food‐web simulator is described in Methods S1, building from Flynn *et al*. (2021). All state variables were described in units of mgN m^−3^. All particulate components were associated with an equivalent spherical diameter (ESD); particle mass was calculated using an allometric equation as per Flynn *et al*. (2021). Encounters between particles depend on their inter‐particle distances, which for organisms is a function of their size (and hence biomass) and the availability of the nutrient that limits their growth; Fig. S1 shows these distances assuming only a single phytoplankton cell of a stated size grown to exhaustion of the nutrient, with equal cell–cell spacing. The suspended sediment particles were described with reference to numerical abundance and their size. Adsorbance of viruses occurred onto all surfaces with the exception of other viruses, so the virus load was affected by the abundance and sizes of all particles, be they organisms, debris or sediment. The zooplankton functional group sub‐model was a development from Mitra (2006); Motility (where enabled and linked to allometry as 3·ESD s^−1^), the biomass‐specific encounter rates and grazing functions were as described in Flynn & Mitra (2016). The food‐web simulator was built and run within Powersim Studio v.10 (https://powersim.com/), as a set of ordinary differential equations under an Euler integration routine with time‐step size of 0.03125 d (= 45 min).

To compare the long‐term competitive advantages between different phytoplankton hosts under different trait configurations, the slopes were calculated from linear regressions (forced through 0,0) fitted through the cumulative production of each phytoplankton type (A1, A2, A3) over a 1000 d simulation period. As actual production values reflect many processes, comparisons are given between the cumulative production of one species vs that of all others in that simulation (total = A1+A2+A3).

### *In silico* experimental scenarios

Experiments were conducted under different nutrient regimes (2.5–40 μM DIN) with motile and nonmotile phytoplankton groups of various sizes (1–20 μm ESD). The BBeD hypothesis was tested with respect to phytoplankton succession with competition and grazing. In all experiments the phytoplankton A1 was the main test species. *In silico* experiments were conducted to explore the competitive advantage of cyst formation by phytoplankton to mitigate viral attack; this was considered by enabling that trait in A1 (cyst cycles of 1 or 3 months) and comparing the outcome with the performance of the non‐cyst‐forming analogue A2, and with total phytoplankton production. Investigations were undertaken with respect to temporal dynamics and long‐term productivities of the phytoplankton of central interest (A1), its non‐cyst‐forming comparator (A2) and the non‐virus‐infected competitor (A3).

## Results

Simulations were run over a wide range of organism sizes and nutrient loading conditions, and with different host trait expressions. We first present some example results, conducted under a few combinations of conditions to demonstrate the temporal dynamics, and then provide summary plots over a matrix of phytoplankton allometry and nutrient loading. All the results shown considered systems containing viruses. In the absence of viruses in the system, A3 goes extinct (as it has a lower growth rate and is thus less competitive) and the temporal dynamics are then of oscillations of the zooplankton predator and its A1+A2 prey (Fig. S2d).

### Trophic dynamics

**Fig. 2:**
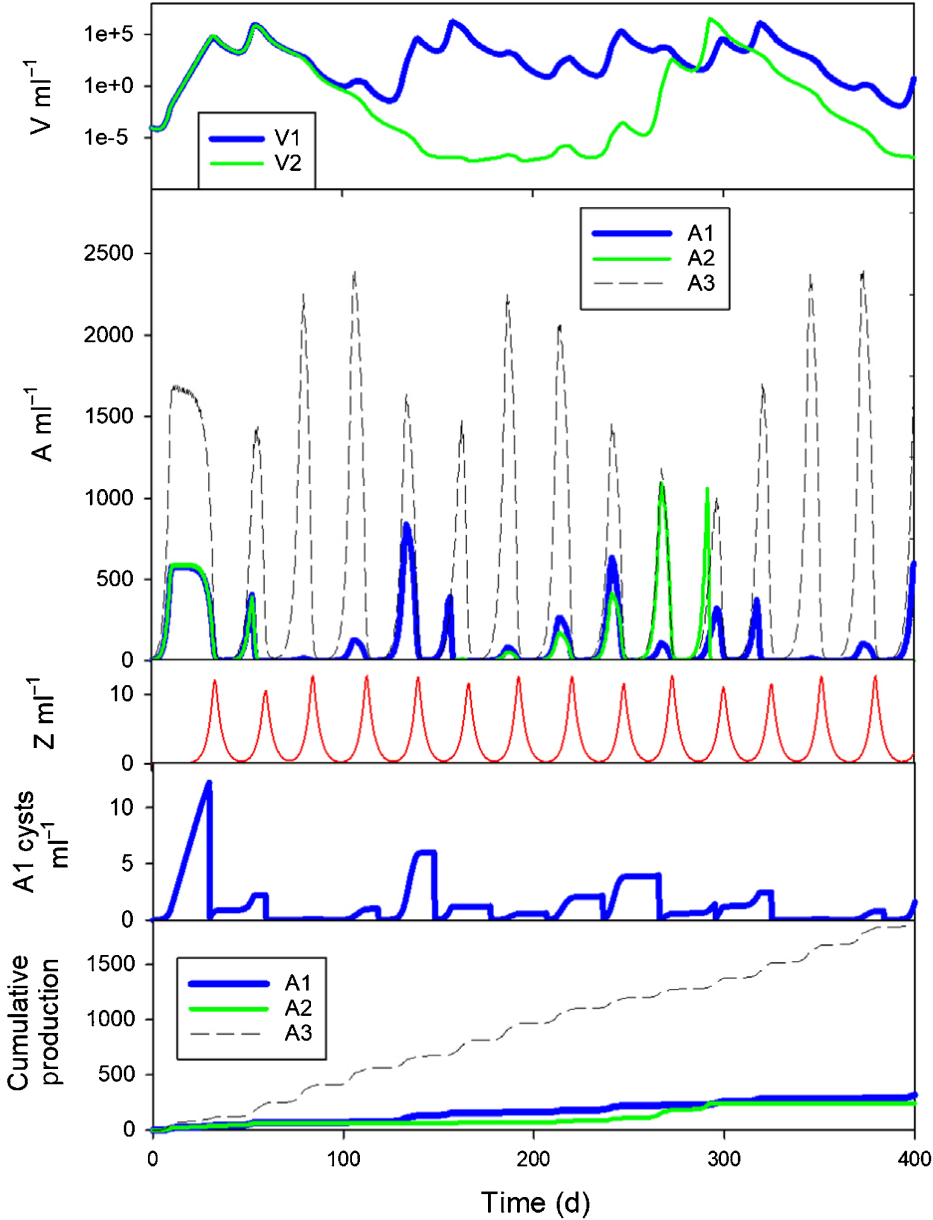
Example output showing the first 400 d of a simulation in which phytoplankton A1, A2 and A3 all have an equivalent spherical diameter (ESD) of 15 µm, grown in a system containing 7.5 µM dissolved inorganic nitrogen (DIN), and with A1 operating a 1‐lunar‐month (29.5 d) cyst cycle. A1 and A2 (and their viruses, V1 and V2) were identical except for the cyst cycles enacted in A1. A3 had no virus but grew with a maximum rate of 90% of that of A1 and A2. The upper panels show abundances for V1 and V2 (grown on hosts A1 and A2 respectively) and the three phytoplankton. The next two panels show zooplankton abundance (Z) and A1 cyst abundance. The bottom panel shows cumulative phytoplankton production (mgN m^−3^); the slopes of linear regressions forced through the origin for each phytoplankton were used in comparisons between production levels. See Fig. 1(b) for a schematic diagram representing the virus‐host–predator system.

Exploration of the temporal dynamics of the coupled virus–host and predator–prey interactions revealed no simple pattern in plankton dynamics. The plots in Fig. 2 show an example model output detailing such dynamics; only the first 400 d of the simulation are shown for clarity. The bottom panel shows the cumulative production of each phytoplankton (A1, A2, A3); the value of these productions at 1000 d was used in considering long‐term consequences (see the next subsection, ‘Long‐term effects of nutrient loading and plankton size’).

The lack of simple repeat cycles of events in Fig. 2 was typical of most simulations. Although long‐duration repeat cycles beyond 1000 d cannot be discounted, in reality abiotic conditions (held constant throughout simulations here) would rarely remain stable for even a few weeks, not least because of changes in the photon flux density and light : dark periodicity. In the model output, it can be seen that the dynamics of total phytoplankton (i.e. A1+A2+A3) growth are constrained by nutrient availability, light levels (via self‐shading by the collective biomass) and grazing. Virus–host dynamics constrained, to a lesser or greater extent, the success of phytoplankton A1 and A2, there being no differential prey selection by Z. In this instance, phytoplankton A1 exhibited a cyst cycle of 1 month duration; cyst cycles of A1 helped to stabilise production of A1 and the abundance of V1 by providing a recurring seed inoculum of hosts. The dynamics of A2 in Fig. 2 are typical for simulated phytoplankton lacking a cyst cycle, showing an increase in host and virus abundances until the virus attack was so great that the host was all but eliminated from the system. Following such an event, it then took many growth cycles for the phytoplankton host and its virus to regain sufficiently high abundance levels to repeat these BBeDs. Thus, we see in Fig. 2 at *c*. 275 d, A2 was dominant over A1 as there was a mismatch between the sequence of success for A1+V1 vs A2+V2. The slower growing, but virus‐free, phytoplankton A3 also produced blooms; these were constrained by nutrient loads (used also by both competitors A1 and A2), and by zooplanktonic (Z) grazing. Comparisons between different combinations of virus inclusion/exclusion and cyst cycles are shown in Fig. S2. This shows the difference in BBeDs (Fig. S2a,b) and the simple oscillatory behaviour of ‘boom‐and‐bust’ predator–prey cycles (Fig. S2c,d).

**Fig. 3:**
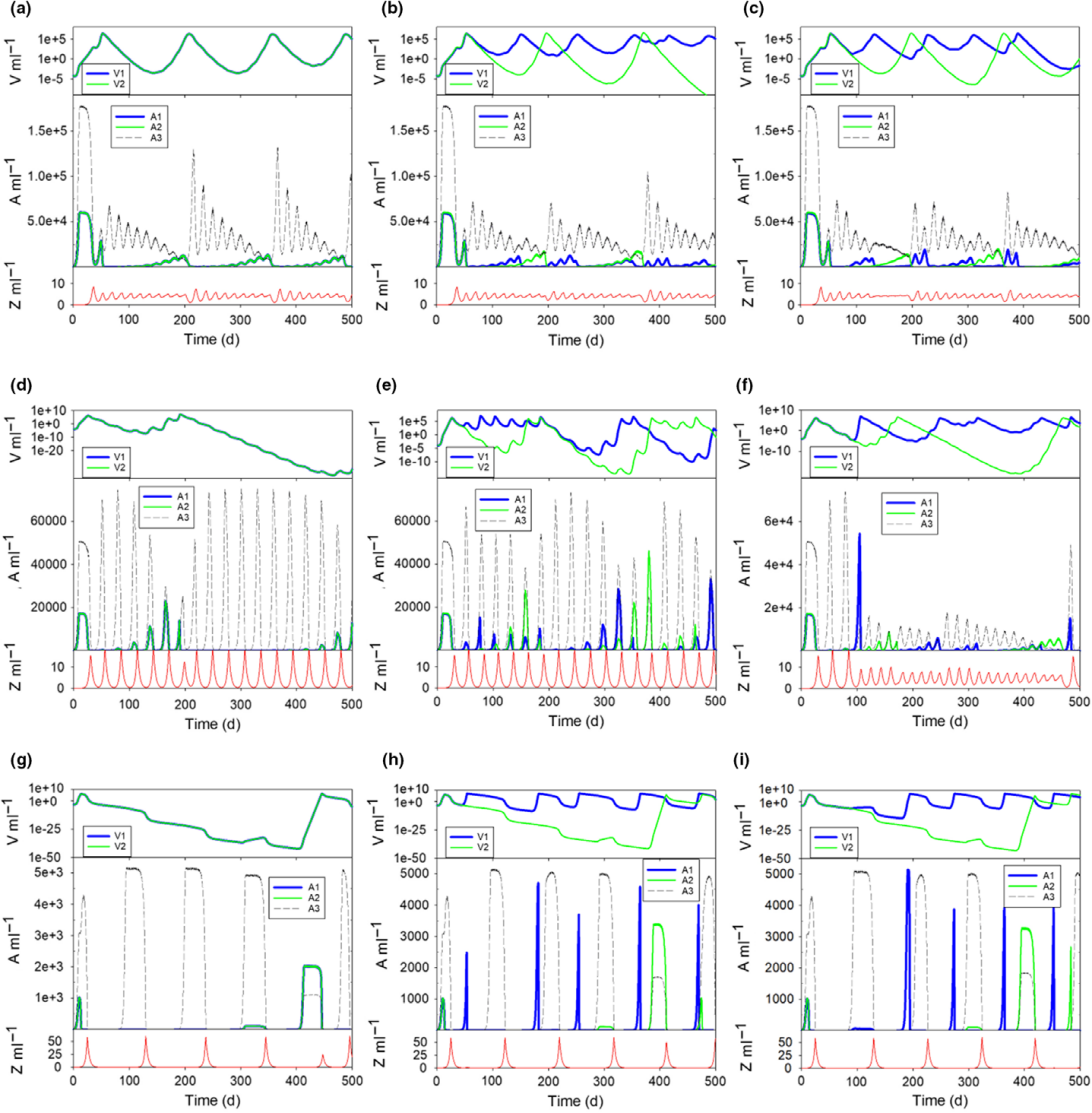
Effects of nutrient loading and organism size on virus–phytoplankton–zooplankton dynamics. Each plot shows the output of the first 500 d of simulations, modifying size, nutrient loading and cyst cycle duration. (a–c) Dynamics under oligotrophic nutrient conditions (5 µM dissolved inorganic nitrogen (DIN)), in which all phytoplankton (A1, A2, A3) have an equivalent spherical diameter (ESD) of 2.5 µm. (d–f) A system akin to mesotrophic conditions (10 µM DIN), in which all phytoplankton (A1, A2, A3) have an ESD of 5 µm. (g–i) Simulations of eutrophic conditions (30 µM DIN), in which all phytoplankton (A1, A2, A3) have an ESD of 20 µm. Phytoplankton A1 is configured with no cyst cycle (a, d, g), a 1‐month cycle (b, e, h) or a 3‐month cycle (c, e, i). In all panels, traits of A1 and A2 (and their viruses, V1 and V2) are identical except for cyst cycles, which can only be enacted in A1.

Different combinations of nutrient loading, phytoplankton cell size and A1 cyst cycle durations induced no clear pattern in the temporal dynamics of the virus–host–predator–prey interactions. Examples of different combinations are shown in Fig. 3. With small‐celled hosts in low nutrient oligotrophic systems (Fig. 3a–c), both virus–host and predator–prey dynamics were moderated by low host or prey biomass levels, respectively. Similar to events shown in Fig. 2, virus–host growth sequences developed where the virus load gradually increased, eventually overwhelming a large bloom of its host. After such an event, both host and virus abundance declined to very low levels for several production cycles, and the BBeD sequence was repeated. With cyst cycles for A1, phytoplankton succession was further altered. Phytoplankton A3 was more successful when A1 and/or A2 were suppressed, but the temporal dynamics of A3 continued to portray a complex appearance, with no simple oscillatory pattern and in some instances with extremes of highs and lows.

Under higher nutrient load (mesotrophic or eutrophic) conditions, the phytoplankton showed protracted BBeD events with bloom sequences that were not the simple oscillations seen in ‘boom‐and‐bust’ events (Fig. 3d–i; cf. Fig. S2d). This was especially so in instances where A1 did not have a cyst cycle (0 month cyst cycle, Fig. 3d,g); phytoplankton A1 and A2, prone as they were to viral attacks, were effectively removed from the system for prolonged periods following very successful bloom events. This was because the virus–host interaction developed to provide virus loads that eventually all but eliminated the host, and thence subsequently also the virus, for several growth cycles.

These BBeD events were less severe when cyst cycles were enabled for A1, although the duration of the cyst cycle had an important impact on dynamics and on the success of A1. With larger cells and higher nutrient input (Fig. 3g–i), in the absence of a cyst cycle, virus production essentially eliminated the host; with no effective reinfection cycles, the virus population itself then decayed to very low levels, sustained only by the continual mixing in of a small residual virus inoculum (Fig. 3g). The predator–prey cycle also showed protracted oscillations (cf. Fig. 3c,f,i). With the inclusion of cyst cycles for A1 the production sequences changed, interestingly not only for A1, but also for A2 (which did not have a cyst cycle) because of changes in resource competition and nonhost adsorption of the viruses. As noted before (e.g. Fig. 2), with cyst cycles, virus V1 was maintained at a higher abundance than was V2 because of the predictable reseeding of the A1 host vs the extreme oscillations of A2.

### Long‐term effects of nutrient loading and plankton size

**Fig. 4:**
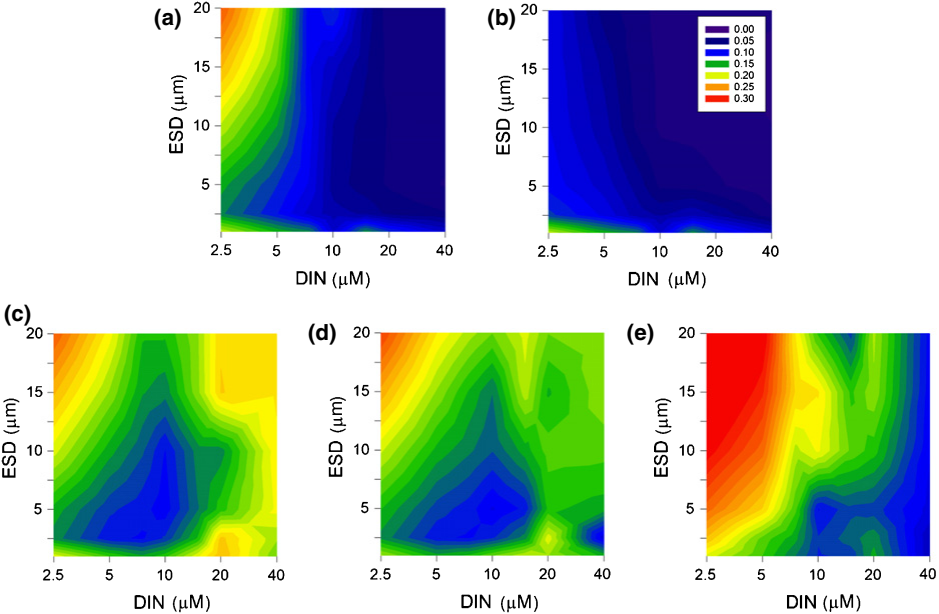
Long‐term effects of nutrient loading and phytoplankton size on the relative success of A1. The overall effect is illustrated in the form of contours which represent the cumulative production of A1 as a fraction of the total phytoplankton production over simulations of 1000 d. All phytoplankton (A1, A2, A3) had the same equivalent spheric diameter (ESD) for each simulation for panels (a–d); the smallest size was 1 µm. DIN, dissolved inorganic nitrogen. (a) Proportion of total cumulative production achieved by A1, where all phytoplankton were nonmotile. (b) Simulation in which A1 and A2 were motile; motility was not enabled for cells with ESD < 2 µm. (c, d) Simulation with nonmotile phytoplankton (cf. (a)) showing the effects of cyst cycles of 1 or 3 months duration, respectively, on the competitive advantage of A1 (see also Supporting Information Fig. S3). (e) As per (a), but here the ESD of A3 was fixed at 1 µm irrespective of the size of A1 and A2.

We further examined the long‐term effects of nutrient loading, phytoplankton size and different traits on the relative success of A1, A2 and A3. Nutrient loading and plankton allometry had coupled effects on virus–phytoplankton dynamics because systems which operated at lower nutrient loads instilled greater distancing between organisms as phytoplankton abundance was decreased (assuming equal spacing between individuals), and the distance is also greatly affected by cell size as smaller cells have a much lower nutrient cell content. The plot in Fig. S1 shows these distances assuming only a single phytoplankton cell of a stated size grown to exhaustion of the nutrient, with equal cell–cell spacing. The consequence of this social distancing of hosts was that larger host cells were less impacted by virus infection at low DIN (Fig. 4a). However, host motility totally removed this advantage (Fig. 4b); motile host cells (not applicable to picophytoplankton, i.e. cyanobacteria, < 2 µm) were much more likely to encounter a virus.

Imposing cyst cycles on phytoplankton A1 (which was otherwise identical to A2, and shared identical virus traits between V1 and V2) improved its production success (Fig. 4c,d). That improvement was most marked at high nutrient loads, where the shorter social distances (Fig. S1) otherwise supported more successful virus infections. A longer cyst cycle (3 months vs 1 month) altered the patterns but was less advantageous to A1 (Fig. S3) as the period between re‐inoculations was longer and BBeD events were more likely. As seen for A1 in Fig. 2, the imposition of cyst cycles of these durations promoted living‐with‐the‐virus events, where the virus was always present at levels that could prevent large blooms but also enabled more stable interactions. The void in phytoplankton biomass left by a virus attack on A1 and A2 offered succession opportunities for other organisms not prone to viral infection, in this instance A3; cyst cycles for A1 also affected the slower growing A3 (Fig. S4). In instances where A2 escaped virus control, it grew faster than A3 (Figs S5, S6). Between A1 and A2 phytoplankton, the survival and succession advantage was with the cyst‐forming A1 (Fig. S7).

There was another allometry‐linked event that is visible in Fig. 4; the size spectrum of the whole community also impacted the virus–host dynamics. In the simulations shown in Fig. 4(a) all phytoplankton (A1, A2, A3) were accorded the same size in any one simulation, and it can be seen that scenarios with the very smallest cells achieved higher A1/total production values than scenarios with a slightly larger size. This was a result of increased adsorption of viruses onto the far more numerous, smaller, nonhost cell particles. Accordingly, re‐running the simulations for Fig. 4(a) but now with A3 fixed at 1 µm ESD in all simulations, mitigated against virus infection in simulations with larger celled A1 and A2 (Fig. 4e).

Debris fragments, similar to an abundance of small organisms, also provide surfaces for the adsorbance of viruses. The number of debris fragments generated by virus‐induced lysis of host cells at burst was set to a default value of 1 (of the same ESD as the host cell). Simulations where the fragment count was related to ESD^0^ (i.e. the default), ESD^2^ (relating to membrane surface area) or ESD^3^ (relating to cell volume) show scope for some interaction with host allometry (Figs S8, S9). It should be noted that such virus adsorptions did not help mitigate against the current virus attack, but they did help mitigate against subsequent infections by lowering the virus load that would act as a future inoculum. For A1, the ESD^3^ result is not as good as that obtained for ESD^2^ (Fig. S9) because adsorption is affected more by particle size than by abundance.

### Long‐term effects of different phytoplankton traits

**Fig. 5:**
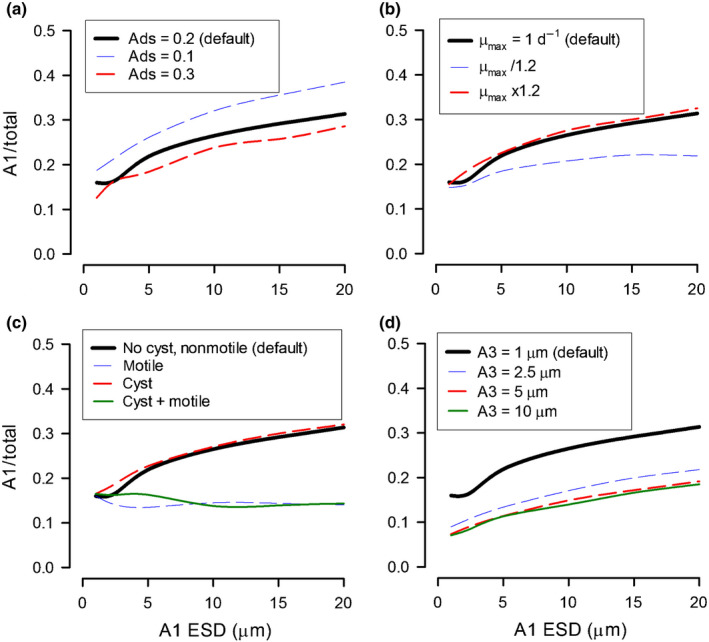
Long‐term effects of phytoplankton size and selected traits on the relative success of A1. Simulations were run at a dissolved inorganic nitrogen (DIN) concentration of 5 µM for 1000 d; ‘Default’ is the 5 µM DIN slice from Fig. 4(e) for a nonmotile, nonencysting A1 species. A2 is identical to A1 in all ways except for cyst cycle dynamics, where applicable. (a) Effect of different virus adsorbance coefficients (‘Ads’), common to all particles other than viruses. (b) Consequences of increasing or decreasing the growth rates (*µ*_max_) of all organisms 1.2 fold. (c) Effects of combinations of cyst cycle and motility. (d) Effect of community allometry; in ‘Default’ and in all simulations for (a–c), A3 has an equivalent spherical diameter (ESD) of 1 µm.

The impacts of virus–phytoplankton interactions on plankton succession with combinations of different phytoplankton traits (e.g. motility, growth rate) were explored (Fig. 5).

*In silico* experiments with A1 and A2 cells of different sizes (but with A3 set constant at an ESD of 1 µm), with DIN at 5 µM and a single globally applied virus adsorption coefficient (default value of 0.2, so 20% of collisions between a virus and another particle resulted in adsorption) provide a reference (Fig. 5a). Decreasing this coefficient increased the success of the susceptible host, and vice versa (Fig. 5a); adsorbance onto hosts in these scenarios had a greater impact than adsorbance onto nonhost particles. Decreasing the plankton growth rates (e.g. simulating growth at a lower temperature) decreased the success of the susceptible host (Fig. 5b); this was not because of a direct virus interaction, but because when the host abundance was not controlled by the virus, it was less likely to achieve high biomass levels before competition and grazing control became significant. While motility enhanced virus infection of the host, damaging production (Fig. 4a,b), and cyst cycles improved production (Fig. 4c,d), the improvement derived from cyst cycles could not compensate for motility (Fig. 5c). The role of the allometry of the community (previously shown in Fig. 4a,e) can be seen in more detail in Fig. 5(d), where progressively increasing the size of A3 negatively affected the success of virus‐susceptible competitor phytoplankton.

### Long‐term grazing impacts

**Fig. 6:**
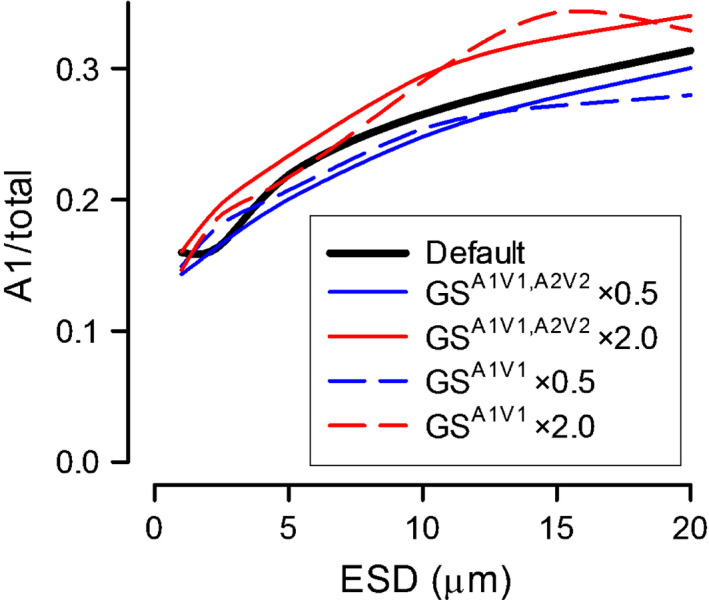
Long‐term effect of phytoplankton size with different levels of grazing on infected hosts on the relative success of A1. Simulations were run at dissolved inorganic nitrogen (DIN) 5 µM for 1000 d. ‘Default’ is the 5 µM DIN slice from Fig. 4(e) for a nonmotile, nonencysting A1 species. A2 growth and infectivity is identical to A1 in all ways. Blue and red lines indicate situations in which grazing selectivity (GS) on infected cells is halved or doubled, respectively, compared to uninfected cells. Solid lines represent simulations in which changes in grazing were applied to both A1V1 and A2V2 (GS^A1V1,A2V2^); dashed lines represent simulations in which grazing changes were only enacted on A1V1 (GS^A1V1^). ESD, equivalent spherical diameter.

All the simulations described thus far assumed that grazing showed no preference for any phytoplankton type, whether it was infected or not (grazing was linked only to biomass‐encounter rates for each prey type). Increasing the capture coefficient of infected hosts enabled the simulation of a situation in which infected phytoplankton were more attractive to grazing and/or such prey resisted capture less; these simulations showed that an increased capture coefficient led to an increased production of the susceptible organism (Fig. 6).

This reflects the loss of virus progeny before host burst. Conversely, if the coefficient was decreased, so that infected cells were rejected as being of poorer quality feed, then production of the susceptible organism decreased. The significance of such selective grazing appears greater for larger prey (Fig. 6), which as a community produced fewer viruses (larger burst size but fewer actual hosts) and hence were more susceptible to changes in the virus abundance loads. Any difference between the changes in grazing being a general response on virus‐infected phytoplankton (solid lines in Fig. 6) rather than a species‐specific effect (dashed lines in Fig. 6) was not clear.

## Discussion

The role of viruses in phytoplankton ecology has hitherto been viewed as a terminator of blooms (Bratbak *et al*., 1993; Tarutani *et al*., 2000; Brussaard & Martinez, 2008; Schatz *et al*., 2014), diverting primary production away from the food web that also supports the biological carbon pump, and into dissolved organic matter, which supports the microbial carbon pump (Wilhelm & Suttle, 1999; Jiao *et al*., 2010; Polimene *et al*., 2017). Our previous work (Flynn *et al*., 2021) revealed that the virus–host dynamic for lytic viruses infecting phytoplankton was critically dependent on the abundances of both parties at the start of the host bloom. Here we see how such interactions, played out in a more complex trophic setting, lead to a series of important cascading effects for phytoplankton succession in community ecology. Specifically, we see a role of viruses in modulating the growth of otherwise potentially highly competitive species (controlling the potential winner in future bloom events), displaying boom‐and‐busted‐dynamics (BBeDs) that help explain the paradox of the plankton. This represents a significant shift in the way that we view the dominant role of viruses in phytoplankton ecology from being a terminator of blooms to being a controller of bloom initiation that has intergenerational and inter‐species consequences.

### Viruses, the paradox of the plankton, and the boom‐and‐busted dynamics hypothesis

Hutchinson's (1961) seminal work on the paradox of the plankton drew attention to the fact that nature does not run to steady‐state. He suggested that inherent oscillations in growth dynamics explained why there are so many plankton species inhabiting what appears, at first sight to humans, to be a rather featureless ecosystem that lacks the niches required to support diversity. While we know that, in reality, micro‐niches, microturbulence and changing light fields make this ecosystem far from featureless (Falkowski, 1984; Fogg, 1991; Reygondeau *et al*., 2013; Behrenfeld *et al*., 2021), our results reveal that viruses contribute to plankton coexistence through the boom‐and‐busted‐dynamic. Indeed, asynchronous controls of different members of the community (Figs 2, 3) provide a mechanism for generating temporal and spatial diversity in the phytoplankton, which in turn provides an explanation for the paradox of the plankton (Hutchinson, 1961) even when abiotic conditions are constant. Not only is the host population affected, but so too are others in the community, competing as they are for common nutrients, and being subject also to grazing. In the real world, abiotic conditions are not constant, and other biological factors (such as predators at higher trophic levels) will also affect the BBeDs. Some changes (such as an input of sediments which absorb viruses – Maat *et al*., 2019) may shorten the expression of the BBeD, making it more closely resemble boom‐and‐bust dynamics.

There is an important interaction between nutrient loading and organism allometry. The higher the nutrient load, the higher the organism numerical abundance, and potentially the larger the size range of phytoplankton (Flynn *et al*., 2018b). These factors together affect the proximity of hosts (Fig. S1), but also the abundance of nonhost particles that remove viruses by adsorption. Larger cells are more susceptible to attack (Murray & Jackson, 1992); while this is moderated by their lower abundance (Fig. 4a), attack is exacerbated by motility (Fig. 4b). However, a numerical predominance of small cells in a population can provide adsorption surfaces that may give protection for those larger organisms present in the same water column (Fig. 5d). We may expect there to also be advantages in being cryptic, as again the summed presence of other organisms and particles associated with food web activity will remove viruses, aided by the greater inter‐host distance afforded by having a low abundance. Virus‐induced BBeD events thus have an allometric component linked to the community structure. There will be additional interactions when one considers that the adsorbance coefficient of different viruses onto different types of particles (assumed constant here, see Fig. 5a) will certainly not be constant in reality.

### ‘Kill‐the‐winner’ vs the boom‐and‐busted hypothesis – the role of viruses in structuring plankton communities

Natural cycles of plankton growth dynamics are highly complex. We tend to either see the collective consequence of phytoplankton growth (most obviously in satellite images) or note extreme events that occur when conditions align in such a way as to allow the rapid growth of an individual species. Traditionally large bloom events have been linked to an escape of phytoplankton growth from grazer control (Irigoien *et al*., 2005; Mitra & Flynn, 2006). However, our simulations show that there is also an important role for virus–host interactions (e.g. Figs 2, 3). In models of plankton ecology, species are rarely portrayed explicitly. More often, whole swaths of often rather distantly related organisms are grouped according to perceived ecological function (e.g. Leles *et al*., 2021). To control the growth dynamics of different populations in traditional models, either a clear limiting resource that differentiates competitive advantage (such as silicon (Si) for diatoms), or some form of grazing function to ‘kill the winner’ (Thingstad, 2000; Winter *et al*., 2010) is required. The problem with the KtW concept is that predators are very rarely (if ever) so selective that only the winner will be grazed preferentially, and on the contrary, selectivity accords with prey palatability such that the dominant species may be completely excluded from grazing, then forming what may be described as an ecosystem disruptive bloom (e.g. Mitra & Flynn, 2006). Most grazing is also allometrically scaled (Allan *et al*., 1977; Flynn *et al*., 1996; Flynn & Mitra, 2016; Tiselius & Møller, 2017), so differential grazing on phytoplankton prey organisms depends on grazer community structure. Viruses, however, have the potential to not only knock out the ‘winner’ but, more importantly, prevent the emergence of the potential winner over the long term as a consequence of the BBeD (Figs 2, 3).

### Virus–phytoplankton–zooplankton trophic dynamics

The intergenerational effects of the BBeD can only be readily studied using simulations, operating as they do over many hundreds of days. Food‐chain interactions in nature are highly complex, with variations in abiotic conditions additionally affecting biotic interactions (Polimene *et al*., 2015). In models under constant environmental conditions, zooplankton–phytoplankton predator–prey dynamics often settle into a repeating oscillatory state (e.g. Smith & Slatkin, 1973; Abrams, 2000; Flynn *et al*., 2018a), but in nature, variations in weather and hydrodynamics inevitably impart a seasonal and more frequent forcing of events. Interactions between virus and grazer controls on common host/prey species are thus modulated by many factors affecting the match and mismatch of abundances. A phytoplankton bloom only develops when a loophole in the control of a species by viruses and grazers occurs together, under conditions that are less favourable for competitors. However, the mode of action of a virus infection is very different to the activity of grazers. The predator has different prey options and can switch between prey types if one species is unavailable (Flynn *et al*., 1996; Flynn & Mitra, 2016). The virus–host couple is instead typically highly specific (Short, 2012) and the extremely high rates of virus propagation can lead to a situation where both parties can tend to extinction (e.g. Fig. 3d–i). A large phytoplankton bloom is thus terminated rapidly, leaving a large virus population which can prevent a substantial repeat host bloom for a long period; this is the BBeD. Eventually, however, the virus control becomes sufficiently weak (due to lack of hosts, adsorption, decay and mixing out) that the host phytoplankton commences a recovery, escaping through the loophole of virus control (cf. blooms developing through the loophole of grazer control – Irigoien *et al*., 2005). This starts with a series of low‐amplitude blooms, simultaneously stimulating a gradual increase in virus numbers, and eventually leading to a large phytoplankton host bloom again and a BBeD event. This sequence of BBeD events is also strongly impacted by other dynamics, such as predator–prey interactions and the growth of competitor phytoplankton, which also affects the nutrient and light conditions that support host growth (Figs 2, 3). Cumulatively, these events can prevent the establishment of a host bloom of sufficient size to start loading the water with viruses. Consistent with the results from our simulations, high biomass blooms of phytoplankton affected by viruses thus appear ephemeral (Suttle *et al*., 1995) and can be extremely difficult to predict.

Using an *in silico* food‐web simulator (Fig. 1), we see that the presence of viruses imparts a quasi‐chaotic appearance upon predator–prey cycles (Figs 2, 3). The dynamics of virus–host interactions played out in a food‐web setting appear to be more repeatable (quasi steady‐state) when operating at low nutrient levels (e.g. Fig. 3a). This set of conditions is representative of oligotrophic waters far from coastal settings, where there is less sediment, which can play a critical role in removing viruses (Maat *et al*., 2019; Flynn *et al*., 2021). These systems are generally more stable abiotically, and under such conditions relatively few species, and their viruses, may be expected to dominate (Cochlan *et al*., 1993; Danovaro *et al*., 2011; Wigington *et al*., 2016; Edwards *et al*., 2021). The very smallest picophytoplankton, the nonprotist (prokaryote) non‐cyst‐producing *Prochlorococcus* and *Synechococcus* (Görl *et al*., 1998; Roth‐Rosenberg *et al*., 2020), dominate these systems; they achieve interhost distancing by growing in low nutrient systems.

Outside of oligotrophic waters, towards the shelf and coasts, we expect more complex dynamics and a wider range of different phytoplankton species and viruses. Here there are additional possible trophic interactions in virus–host/prey–predator systems. Grazers may directly remove viruses (González & Suttle, 1993), faecal pellets may provide vectors for the transfer of viruses from ingested prey to different water patches (assuming the viruses de‐adsorb), and then there is the issue of whether infected hosts are more, or less, favoured as prey items (Evans & Wilson, 2008; Vermont *et al*., 2016). Protozooplankton in particular can display changes in preference for prey on account of food quality, even for a single prey species (Mitra & Flynn, 2006; Mitra, 2006). A virus‐infected host may be expected to release a different suite of organics (‘smell’ different), affecting prey selectivity (Martel, 2006). That and any differences in behaviour, such as swimming, may well be expected to affect selection for predation one way or the other. However, our simulations did not reveal a marked consequence for virus–host dynamics by invoking either positive or negative discrimination in grazing. Any effect seems most evident at larger host cell sizes (Fig. 6); these populations in the simulations produced far fewer viruses, and so any changes in losses of viral progeny could be more significant. Larger hosts may also produce more fragments on bursting, which may have a limited impact on subsequent virus loading (Fig. S9).

We may also expect less impact of grazing on infected phytoplankton hosts with shorter latent times (at elevated temperatures, or with higher growth rates), as the window for any differential grazing to be enacted upon an individual infected cell will be shorter. Virus replication is a function of host growth rate (so a slower growing infected host would produce viruses more slowly as well; reworking of the data in Edwards *et al*., 2021; Flynn *et al*., 2021); the explanation for why a slower growth rate apparently favoured virus attack (i.e. A1/total was decreased in Fig. 5b) is thus a reflection of the change in predator–prey dynamics in systems in which the prey did not grow so rapidly. Larger, slower growing cells may be more likely to be controlled by viruses, assuming all else is equal. Changing (increased) temperatures are expected to have radical impacts on whole‐system trophic dynamics (Gaedke *et al*., 2010; Calbet *et al*., 2014), which will exert more fundamental pressures on the potential for and consequences of virus–host interactions.

### Spatial and temporal distancing

Virus–phytoplankton interactions are less likely when the inter‐host distance is greater, and when periods between infection events are longer. Competitive advantage evolves over many hundreds of generations, with each of those generations experiencing highs and lows of local success depending on biotic and abiotic conditions. Here we explored the generality of trait success by reference to the cumulative production (which mirrors the number of cell replications). In general, larger phytoplankton hosts, and lower nutrient loads promote distancing between hosts (Fig. S1), decreasing the likelihood of further infection set against a decay in virus abundance over time (Fig. 4). This trend is enhanced with faster growth, but importantly is nullified if the hosts are motile (Figs 4b, 5c), noting that larger motile cells typically swim faster (Flynn & Mitra, 2016). This suggests that motile and typically slower growing groups are more susceptible to viral control (Fig. 6; Flynn *et al*., 2021). By contrast, under similar nutrient loadings, larger celled, nonmotile phytoplankton, such as diatoms, would be relatively safe from further virus attack, mainly because they are likely already infected by hidden (or latent) infections that do not kill the hosts until the Si supply has been exhausted (Kranzler *et al*., 2019).

For cyst cycles to protect phytoplankton from viral attack, there must be very few hosts remaining in circulation, and yet the total removal of a population to cysts also prevents population growth and provides competitors with opportunities. We found that having short‐term cyst cycles, while being generally good for the host through decreasing the virus load for subsequent bloom development, also leads to cycles of re‐establishment of the host and virus in broad synchrony. This results in ‘living‐with‐the‐virus’ events (akin to the situation seen for the prokaryote picoplankton in low nutrient systems; A1 vs A2 in Figs 2, 3).

Here the cyst cycles of 1 or 3 lunar months not only provided a refuge for host populations, ensuring a relatively good inoculum for when favourable conditions arose, they also provided a situation that prevented extremely low virus numbers. This stabilises the system, giving rise to relatively low‐amplitude oscillations consistent with the appearance of background or cryptic phytoplankton species. By contrast, the absence of cyst cycles, especially for larger cells at higher nutrient concentrations, gives rise to strong oscillations in abundance which could be interpreted in nature as an occasional introduction of a dominating species when actually it reflects an occasional escape from virus control as an example of an extreme BBeD event (Fig. 3g–i). Such extreme dynamics in nature could be caused by rare mass excystment of the host, against a background of vanishingly low viral loads. If that occurred in coastal waters loaded with sediment, which can remove viruses (Maat *et al*., 2019; Flynn *et al*., 2021), then virus control is even less likely. Either way, we can see from the complex nature of the dynamics even in a stable abiotic setting that there is a clear potential for outwardly unpredictable one‐off bloom events (Figs 2, 3).

### Future work

Exploration of the factors related to the paradox of the plankton has long provided a challenge for modellers (Petersen, 1975; Huang *et al*., 2016). Our work, as the first attempt to produce a complex description of virus–host plankton interactions in an ecological setting, demonstrates a role for viruses as enablers or disablers of phytoplankton bloom dynamics that is more profound than previously thought. How we proceed with simulating the role of viruses in phytoplankton ecology as a consequence of BBeDs is as yet unclear, and it should be noted that virus dynamics are in any case typically poorly described in ecosystem models (Mateus, 2017). Our results highlight the importance of better understanding the factors that control the loss of viruses from the water column, and how host phytoplankton maximise spatial and temporal distancing to minimise infection while still maximising their own reproduction. Applications in models are likely to be of most relevance for the simulation of species which exhibit spectacular growth rates and are known to be affected by viruses, such as *Emiliania huxleyi* (Bratbak *et al*., 1993; Wilson *et al*., 2002), cosmopolitan species (*Prochlorococcus*, Sullivan *et al*., 2003; *Synechococcus*, Suttle & Chan, 1994; *Chrysochromulina*, Suttle *et al*., 1995), and organisms deemed to be harmful or ecosystem disruptive (*Aureococcus*, Milligan & Cosper, 1994; *Phaeocystis;* Jacobsen *et al*., 1996). To support such efforts requires appropriate data for the meaningful modelling of whole‐ecosystem dynamics.

## Author contributions

AM, KJF and WHW planned and designed the research. KJF and AM developed the *in silico* simulator, performed the experiments and conducted visualization. AM, KJF, WHW, SAK, DRC, AP and LP contributed towards data interpretation and the writing of the manuscript.

## Supporting information


**Fig. S1** The average cell–cell distance for cells of different sizes at different biomass abundances.
**Fig. S2** Different dynamics achieved by applying combinations of cyst cycles to A1, and virus infection to A1 and A2.
**Fig. S3** Percentage differences in long‐term productivity of phytoplankton A1 resulting from cyst cycles of different durations.
**Fig. S4** Long‐term effect of A1 phytoplankton cyst cycles of different durations on productivity of A3 phytoplankton.
**Fig. S5** Long‐term effect of phytoplankton A1 cyst cycles of different durations on phytoplankton A2 productivity.
**Fig. S6** Percentage differences in long‐term productivity of phytoplankton A2 resulting from phytoplankton A1 cyst cycles of different durations.
**Fig. S7** Competitive advantage between phytoplankton A1 and A2 when one of them (A1) can encyst with a 1‐month cyst cycle or a 3‐month cycle.
**Fig. S8** Long‐term effect of nutrient loading and phytoplankton size on the relative success of phytoplankton A1, considering different fragmentations of A1 cells on bursting.
**Fig. S9** Percentage differences in the long‐term productivity of phytoplankton A1 according to the degree of fragmentation upon A1 cell burst.
**Methods S1** Mathematical constructs.
Please note: Wiley Blackwell are not responsible for the content or functionality of any Supporting Information supplied by the authors. Any queries (other than missing material) should be directed to the *New Phytologist* Central Office.

## Data Availability

The data that support the findings of this study are available in the Supporting Information associated with this article.
