# Supplementary material for: ‘Boom‐and‐busted’ dynamics of phytoplankton–virus interactions explain the paradox of the plankton
Source: New Phytol. 2022 Mar 9;234(3):990–1002. doi: 10.1111/nph.18042 (PMC9313554; doi:10.1111/nph.18042)
Supplement: Supplementary file 1 — Fig. S1 The average cell–cell distance for cells of different sizes at different biomass abundances. Fig. S2 Different dynamics achieved by applying combinations of cyst cycles to A1, and virus infection to A1 and A2. Fig. S3 Percentage differences in long‐term productivity of phytoplankton A1 resulting from cyst cycles of different durations. Fig. S4 Long‐term effect of A1 phytoplankton cyst cycles of different durations on productivity of A3 phytoplankton. Fig. S5 Long‐term effect of phytoplankton A1 cyst cycles of different durations on phytoplankton A2 productivity. Fig. S6 Percentage differences in long‐term productivity of phytoplankton A2 resulting from phytoplankton A1 cyst cycles of different durations. Fig. S7 Competitive advantage between phytoplankton A1 and A2 when one of them (A1) can encyst with a 1‐month cyst cycle or a 3‐month cycle. Fig. S8 Long‐term effect of nutrient loading and phytoplankton size on the relative success of phytoplankton A1, considering different fragmentations of A1 cells on bursting. Fig. S9 Percentage differences in the long‐term productivity of phytoplankton A1 according to the degree of fragmentation upon A1 cell burst. Methods S1 Mathematical constructs. Please note: Wiley Blackwell are not responsible for the content or functionality of any Supporting Information supplied by the authors. Any queries (other than missing material) should be directed to the New Phytologist Central Office. [file NPH-234-990-s001.pdf]

***New Phytologist* Supporting Information**

Article title:
"Boom-and-busted-dynamics" of phytoplankton-virus interactions explain the paradox of the plankton

Authors:
Kevin J Flynn, Aditee Mitra, William H Wilson, Susan A Kimmance, Darren R Clark, Angela Pelusi, Luca Polimene

Article acceptance date:
04 February 2022

The following Supporting Information is available for this article:

Fig. S1 The average cell-cell distance for cells of different size at different biomass abundance.

Fig. S2 Different dynamics achieved by applying combinations of cyst cycle to A1, and virus infection to A1 and A2.

Fig. S3 Percentage differences in long term productivity of phytoplankton A1 in consequence of cyst cycles of different durations.

Fig. S4 Long term effect of A1 phytoplankton cyst cycles of different durations on productivity of A3 phytoplankton.

Fig. S5 Long term effect of phytoplankton A1 cyst cycles of different durations on phytoplankton A2 productivity.

Fig. S6 Percentage differences in long term productivity of phytoplankton A2 in consequence of phytoplankton A1 cyst cycles of different durations.

Fig. S7 Competitive advantage between phytoplankton A1 and A2 when one of them (A1) can encyst with a 1 mo cyst cycle or 3 mo cycle.

Fig. S8 Long term effect of nutrient loading and phytoplankton size on relative success of phytoplankton A1, considering different fragmentations of A1 cells on bursting.

Fig. S9 Percentage differences in long term productivity of phytoplankton A1 in consequence of the degree of fragmentation upon A1 cell burst.

Methods S1 Mathematical construct

FIGURES

Fig.S1 The average cell-cell distance for cells of different size at different biomass abundance. Calculations assume that all dissolved inorganic N (DIN) is converted into biomass of a single phytoplankton species of the indicated equivalent spherical diameter (ESD). Cell carbon was computed from cell nitrogen assuming cellular C:N as per the Redfield ratio. Cell carbon was then converted to ESD using the equations of Menden-Deuer & Lessard (2000). The distance is shown between cell-surfaces assuming a homogeneous distribution. The minimum distance between cells on this plot (at DIN = 40 µM, ESD = 1 µm) is 31 µm.

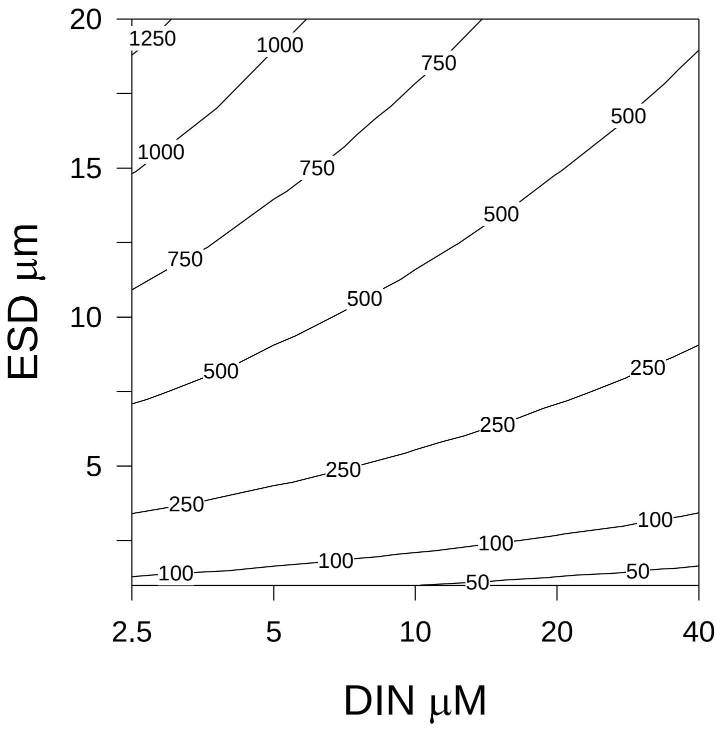

Menden-Deuer S, Lessard EJ. 2000. Carbon to volume relationships for dinoflagellates, diatoms, and other protist plankton. *Limnology and Oceanography.* **45**: 569–579. doi:10.4319/lo.2000.45.3.0569

Fig.S2 Different dynamics achieved by applying combinations of cyst cycle to A1, and virus infection to A1 and A2. The first 400d of panel (a) is the same as Fig. 2 with virus infections enabled for both A1 and A2, and A1 having a 1mo cyst cycle. Panel (b) is the same as (a), but with no cyst cycle; A1 and A2 are thus identical. Panel (c) has no virus but retains a cyst cycle for A1; here A3 rapidly becomes extinct, and A1 also outcompetes A2. Panel (d) has neither virus nor cyst cycles; A3 becomes extinct and A1 is identical to A2. Note that in the absence of viruses (panels (c) and especially in (d)) the dynamics are classic "boom-and-bust" with a regular predator-prey cycle. With viruses (panels (a) and (b)), cycles of phytoplankton growth display "boom-and-busted" dynamics, where a large virus-host event prevents significant host bloom development for some considerable time. DIN - dissolved inorganic N; ESD - equivalent spherical diameter. The legend of Fig. 2 provides more details.

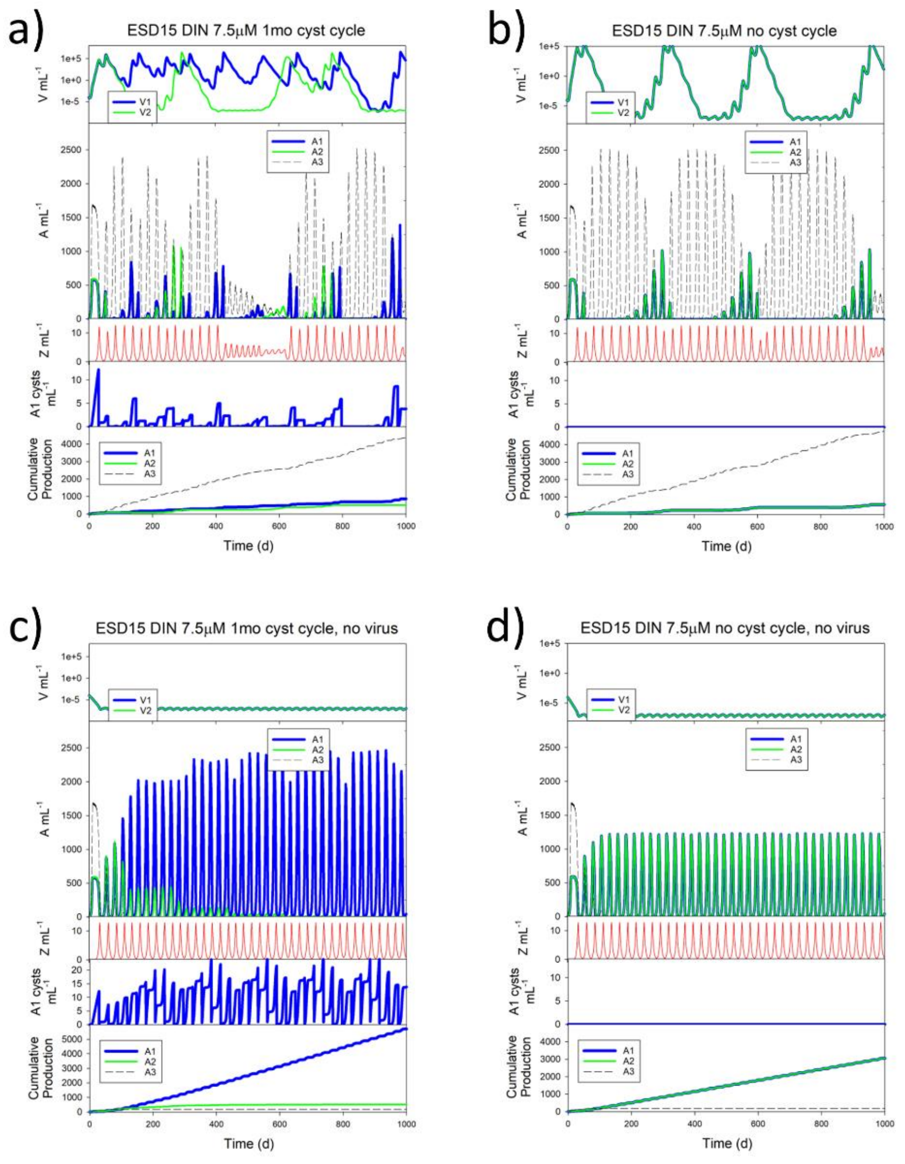

Fig.S3 Percentage differences in long term productivity of phytoplankton A1 in consequence of cyst cycles of different durations. Contours show, in panel (a) the percentage difference between 1 mo cycle and no cyst cycle in A1, calculated as 100·(Fig.4c-Fig.4a)/Fig.4a; in panel (b) the percentage difference between 3 mo cycle and no cyst cycle in A1, calculated as 100·(Fig.4d-Fig.4a)/Fig.4a. Nutrient loading is indicated by the dissolved inorganic N concentration (DIN), with the host size indicated by the equivalent spherical diameter (ESD).

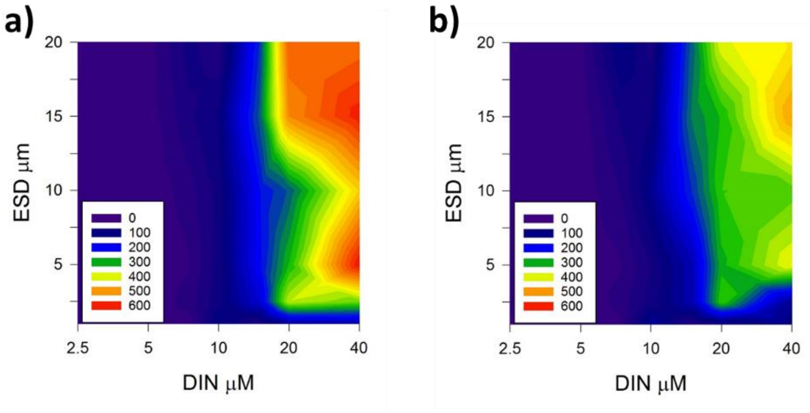

Fig.S4 Long term effect of A1 phytoplankton cyst cycles of different durations on productivity of A3 phytoplankton. This is shown as contours of the proportion of cumulative production of A3 compared to total phytoplankton production over 1000 d simulations (i.e., A3/Total; cf. Fig.S3 for A1/Total). Panel (a) shows impact of no A1 cyst cycle, panel (b) is for 1 mo A1 cycle and panel (c) is for 3 mo A1 cycle. Nutrient loading is indicated by the dissolved inorganic N concentration (DIN), with the host size indicated by the equivalent spherical diameter (ESD).

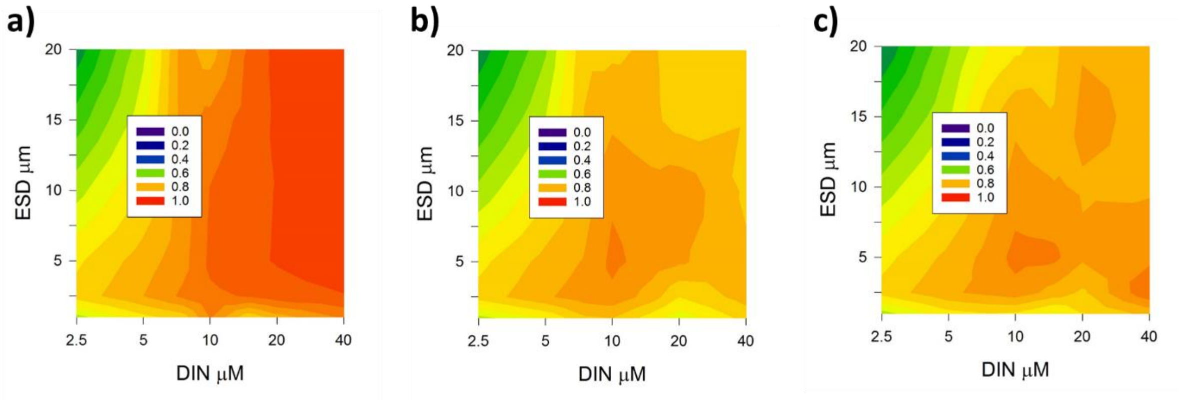

Fig.S5 Long term effect of phytoplankton A1 cyst cycles of different durations on phytoplankton A2 productivity. This is shown as contours of the proportion of cumulative production of A2 compared to total phytoplankton production over 1000 d simulations (i.e., A2/Total). Panel (a) shows impact of no A1 cyst cycle, panel (b) is for 1 mo A1 cycle and panel (c) is for 3 mo A1 cycle. Note that A1 and A2 are identical except for the ability in A1 to encyst; cf. Fig.4a, 4c, 4d respectively for A1/Total. Nutrient loading is indicated by the dissolved inorganic N concentration (DIN), with the host size indicated by the equivalent spherical diameter (ESD). See also Fig.S6 for the enhancement percentages.

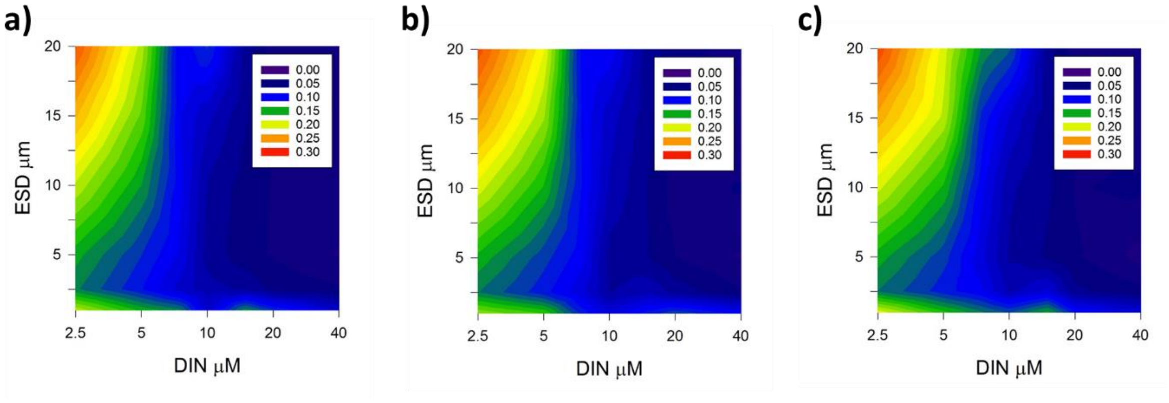

Fig.S6 Percentage differences in long term productivity of phytoplankton A2 in consequence of phytoplankton A1 cyst cycles of different durations. Contours show, in panel (a) the percentage difference between no A1 cyst cycle and 1 mo cycle, calculated as 100·(Fig.S5b-Fig.S5a)/Fig.S5a; panel (b) shows the percentage difference between no A1 cyst cycle and 3 mo cycle, calculated as 100·(Fig.S5c-Fig.S5a)/Fig.S5a. DIN - dissolved inorganic N; ESD - equivalent spherical diameter.

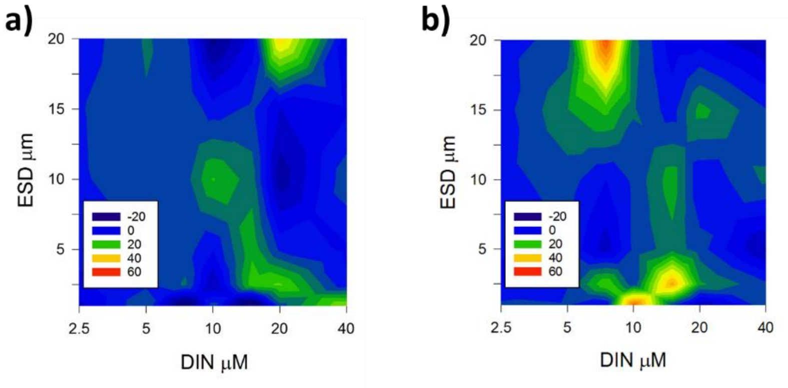

Fig.S7 Competitive advantage between phytoplankton A1 and A2 of otherwise identical traits, both with identical trait viruses, when one of them (A1) can encyst with a 1 mo cyst cycle (panel (a)) or 3 mo cycle (panel (b)). Contours show the ratio of A1:A2 production over 1000 d simulations; thus panel (a) = Fig.4c/Fig.S5b, and panel (b) = Fig.4d/Fig.S5c. Nutrient loading is indicated by the dissolved inorganic N concentration (DIN), with the host size indicated by the equivalent spherical diameter (ESD).

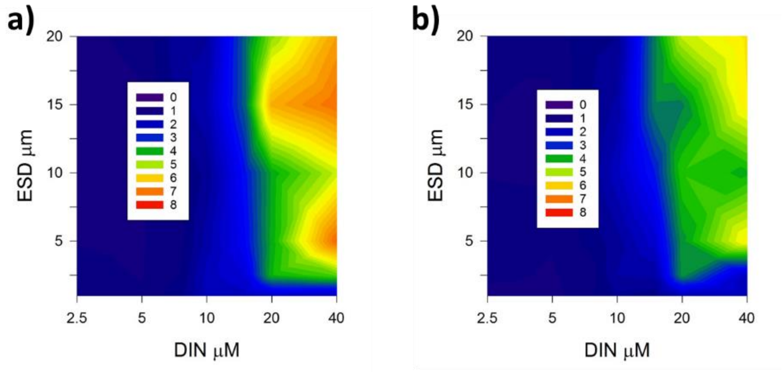

Fig.S8 Long term effect of nutrient loading and phytoplankton size on relative success of phytoplankton A1, considering different fragmentations of A1 cells on bursting. This is shown as contours of the proportion of cumulative production of A1 compared to total phytoplankton production over 1000 d simulations (i.e., A1/Total). All details as for Fig.4a except the number of fragments released per bursting A1 cell, which were set as follows: panel (a), one fragment (i.e., ESD^0, identical to plot Fig.4a); panel (b), related to surface area (ESD^2); panel (c), related to cell volume (ESD^3). ESD - equivalent spherical diameter. See also Fig.S9.

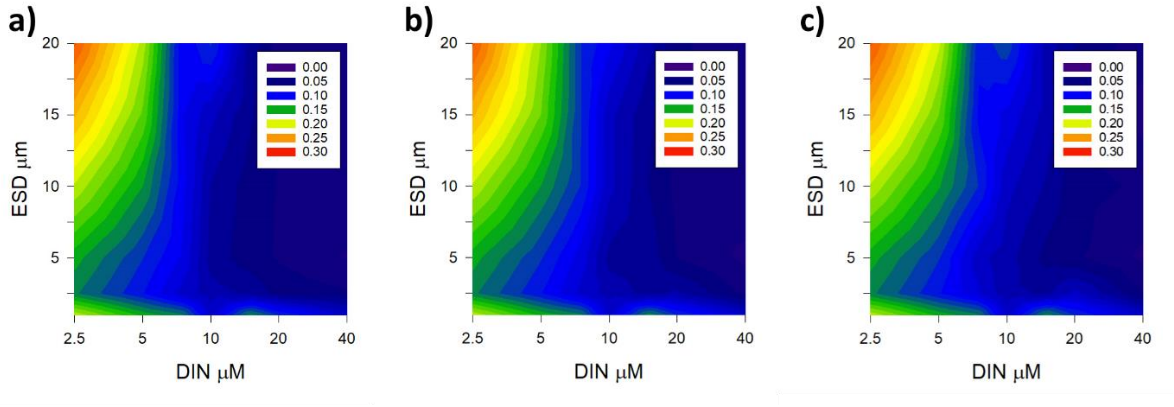

Fig.S9 Percentage differences in long term productivity of phytoplankton A1 in consequence of the degree of fragmentation upon A1 cell burst. Contours show, in panel (a) the percentage difference between ESD^2 and ESD^0, calculated as 100·(Fig.S8b-Fig.S8a)/ Fig.S8a; in panel (b) the percentage difference between ESD^3 and ESD^0, calculated as 100·(Fig.S8c-Fig.S8a)/Fig.S8a. Nutrient loading is indicated by the dissolved inorganic N concentration (DIN), with host size indicated by the equivalent spherical diameter (ESD).

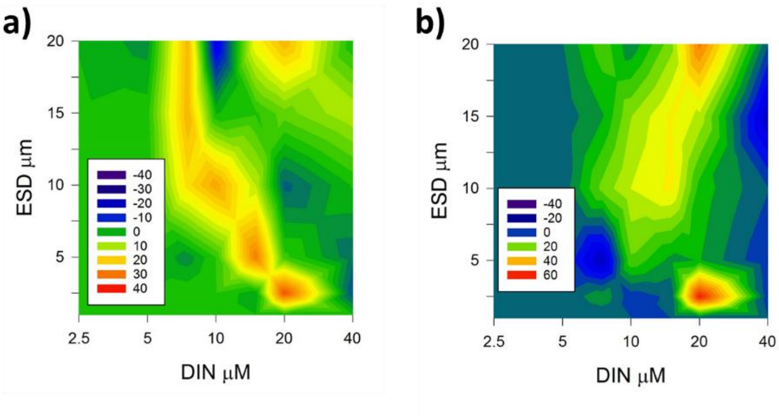

METHODS S1

Equations describing the virus model and the phytoplankton are described in full detail for a single virus-host couple in Flynn *et al.*, (2021); the following gives a discursive overview of the virus-phytoplankton components, noting that here we now had two virus-host couples. See Fig.1b for a schematic of the model.

The virus-host model comprised state variables for:

- inorganic nutrient (DIN, as ammonium),
- host phytoplankton (A1, A2) of a stated equivalent spherical diameter (ESD), maximum growth rate (μmax) and motility (either not motile or with motility allometrically scaled as 3 × ESD s^{-1}; Flynn & Mitra, 2016)
- virus (V1, V2) associated with hosts A1 and A2 respectively, as free particles
- infected A1 and A2 hosts (A1V1, A2V2)
- fragments of burst A1V1 and A2V2

In addition, the model included (as per Flynn *et al.*, 2021):

- an implicit bacterial activity that converted (decayed) all N associated with cell fragments and detritus, and also adhered viral particles, back to DIN.
- suspended inert particles which affected virus-host encounters by adsorbing viruses.
- mixing within a water column of a stated mixing depth which acts in a fashion akin to a chemostat dilution, bringing in nutrient particles from outside of the mixed layer, and washing out a proportion of all materials in the mixed layer.

To the enlarged model of Flynn *et al*. (2021), as per above, we also added:

- cyst stages for A1; into this stage variable was removed a small fraction of A1, with excystment being triggered every 1 or 2 lunar months (1 or 3 × 29.5d).
- an additional phytoplankton (A3) which was described physiologically in exactly the same way as A1 and A2, except that μmax was at 90% of that set for A1 and A2.
- a zooplankton functional group that grazed upon the phytoplankton (A1, A2, A3) and also upon infected phytoplankton (A1V1, A2V2) according to biomass-

specific encounter rates with no prey discrimination as the default setting. This zooplankton submodel was otherwise as described in chapter 5 of Flynn (2018). Regenerated nutrient from zooplankton activity directly entered the DIN pool, while faecal material was degraded in the same way that debris was degraded in the original virus model (Flynn *et al*., 2021).

All state variables were described in units of mgN m^{-3}.

All particulate components were also associated with an equivalent spherical diameter (ESD), and thence with a particle mass calculated from an allometric equation of Flynn *et al*. (2021), following Menden-Deuer & Lessard (2000). The suspended sediment particles were described with reference to numeric abundance and their size.

Functional equations

Functionally, the processes are described as follows:

A1,A2,A3 and A1V1, A2V2 phytoplankton population growth =
f{phytoplankton biomass, DIN, light} Eq.1

Infection by V1 into A1 and V2 into A2 =
f{host abundance, virus abundance, host ESD, virus ESD, host motility, adsorbance, infectivity} Eq.2

Host-virus (A1V1, A2V2) lysis =
f{host-virus growth, latent period, burst size} Eq.3

where burst size was itself a function of the nutrient status of the host and host ESD, and latent period is a function of host growth rate (see Flynn *et al*., 2021).

Lysed host (A1i, A2i) fragment decay =
f{implicit bacterial-mediated decay} Eq.4

Virus (V1, V2) loss and decay =
f{virus adsorbance onto particles, bacteria-mediated decay, UV-decay} Eq.5

Virus adsorbance occurred onto all surfaces other than other viruses, so the virus load was affected by the abundance and sizes of all particles, be they organisms, debris or sediment.

Zooplankton population growth on prey A1, A2, A3, A1V1, A2V2 with assimilation efficiency AE and specific dynamic action SDA =

f{zooplankton biomass, prey biomass abundance, AE, SDA, catabolic respiration}

Eq.6

Simulations and outputs

The model was built and run within Powersim Studio10 (www.Powersim.com), as a set of ODEs under an Euler integration routine with time-step size of 0.03125 d (=45min).

Simulations were run over 1000d, recording the cumulative production of each organism group. The slopes of linear regressions (forced through 0,0) through the cumulative production (cumProd) data were used to compare the relative success of each phytoplankton population to the total. For example, between A1 and the total:

cumProd{A1} / ∑ (cumProd{A1,A2,A3})

or between A1 and A2 as:

cumProd{A1} / cumProd{A2}

Additional References

Flynn KJ. 2018. *Dynamic Ecology - an introduction to the art of simulating trophic dynamics*. Swansea University, Swansea, UK. ISBN: 978-0-9567462-9-0 https://cronfa.swan.ac.uk/Record/cronfa40405

Flynn KJ, Kimmance SA, Clark DR, Mitra A, Polimene L, Wilson WH. 2021. Modelling the effects of traits and abiotic factors on viral lysis in phytoplankton. *Frontiers in Marine Science* **8**: 667184.

Menden-Deuer S, Lessard EJ. 2000. Carbon to volume relationships for dinoflagellates, diatoms, and other protist plankton. *Limnology and Oceanography* **45**: 569–579.doi:10.4319/lo.2000.45.3.0569
